# Review of the Role of HER2/neu in Colorectal Carcinomas

**DOI:** 10.7759/cureus.25409

**Published:** 2022-05-27

**Authors:** Lakshmi Sai Vijay Achalla, Raju K Shinde, Sangita Jogdand, Sahitya Vodithala

**Affiliations:** 1 Department of Surgery, Acharya Vinoba Bhave Rural Hospital (AVBRH) Jawaharlal Nehru Medical College (JNMC) Datta Meghe Institute of Medical Sciences (DMIMS), Wardha, IND; 2 Department of General Surgery, Datta Meghe Institute of Medical Sciences (DMIMS) University, Wardha, IND; 3 Department of Pharmacology, Acharya Vinoba Bhave Rural Hospital (AVBRH) Jawaharlal Nehru Medical College (JNMC) Datta Meghe Institute of Medical Sciences (DMIMS), Wardha, IND; 4 Department of Pathology and Laboratory Medicine, Acharya Vinoba Bhave Rural Hospital (AVBRH) Jawaharlal Nehru Medical College (JNMC) Datta Meghe Institute of Medical Sciences (DMIMS), Wardha, IND

**Keywords:** mcrc, immunohistochemistry, trastuzumab, her2/neu, colorectal cancer

## Abstract

Human epidermal growth factor receptor 2 (HER2/neu) is an oncogenic driver and a proven target for treatment of breast and gastric cancers. The role of HER2/neu and its blockage in various tumors, particularly colorectal adenocarcinoma has been widely explored following the revolutionary impact of anti-HER2/neu therapy in breast and gastric carcinoma patients. This review aimed to highlight the most recent updates on the significance of HER2/neu as a prognostic and predictive factor in these tumors together with its subsequent possible therapeutic indications from preclinical tests and ongoing assessments testing anti-HER2/neu agents in colorectal carcinoma (CRC). In the near future with a growingly tailored therapeutic approach toward cancers, HER2/neu targeted therapeutic strategies may blend into CRC treatment methods.

## Introduction and background

Regardless of the advancements in the treatment protocols and regimens, colorectal carcinoma (CRC) pursues to be one of the prime causes of malignancy-associated mortality worldwide. Further advances in the understanding of tumor biology and identification of oncogenic drivers have resulted in the identification of novel therapeutic targets. As a result, identifying biological indicators for targeted therapy remains an essential goal in the treatment of human cancer. The therapy with anti-epidermal growth factor receptor (anti-EGFR) monoclonal antibodies, which include panitumumab and cetuximab, exhibited to augment the progression-free survival in patients with advanced CRC with wild-type Kirsten rat sarcoma viral oncogene homolog (KRAS) mutations [1,2].

Despite significant therapeutic advancements, a remarkable subset of cancer patients do not respond well to treatment and therapeutic response cannot be anticipated precisely. As a result, it is critical to find molecular biomarkers capable of predicting prognosis, therapeutic response, and possible therapeutic targets for colorectal cancer patients. Trastuzumab is a humanized monoclonal antibody that specifically targets the human epidermal growth factor receptor 2 (HER2/neu) receptor's extracellular domain. Its therapeutic effect has been established in HER2/neu-positive breast carcinomas patients [3,4].

Additionally, a major trial, Trastuzumab for Gastric Cancer (ToGA) trial published in 2010 has shown that combining trastuzumab with conventional chemotherapy notably increased durability in patients with advanced gastric or gastroesophageal (GE) junction carcinomas who were HER2/neu-positive [4]. Amplification of the HER2/neu gene and increased expression of the HER2/neu protein were reported in 20% of breast carcinoma patients which were accompanied by an aggressive phenotype, metastases with a poor prognosis [5].

Amplification as well as increased expression of the HER2/neu gene along with HER2/neu protein was found in 22% of patients with gastric carcinomas, although their prognostic relevance is still debatable. Because trastuzumab has been shown to dramatically increase overall survival in carcinomas of breast and stomach, there is considerable clinical gain in determining if HER2/neu blockage may be a helpful therapeutic strategy in other human malignancies [4,5]. A small number of studies already have described the prevalence as well as clinical implications of HER2/neu status in individuals with colorectal cancer, yet its clinical importance has not been clearly established [6-8].

## Review

HER2/neu as an oncogenic driver in CRC

HER2/neu which is also called ERBB2 is situated on the long arm of chromosome 17q12 and codes 185kd transmembrane glycoprotein receptor. It has an intrinsic tyrosine kinase activity as opposed to alternative family members like HER1, HER3, and HER4. HER2/neu is known as an "orphan receptor" due to the absence of an endogenous ligand. Its activation mainly relies on homodimerization or more commonly heterodimerization with other EGFR receptors, especially HER1 and HER3, consequently proceeding in transphosphorylation of tyrosine residues inside its cytoplasmic domain [9].

Their structure includes a ligand-binding extracellular domain, a transmembrane as well as an intracellular tyrosine kinase domain. The system is very complicated where the tasks of this family are carried out by not less than 12 ligands and four receptors. In short, the signal transduction pathways, including mitogen-activated protein kinase (MAPK), mammalian target of rapamycin (mTOR), phosphoinositide 3-kinase (PI3K), and protein kinase B (AKT), results in cellular expansion, differentiation, hindrance of apoptosis, and cancer progression. This allows HER2/neu activation without ligand binding to other partners. In neoplasia, the pathogens get poorly regulated and cause HER2/neu overexpression, which leads to unrestricted tumor cell expansion and migration [10].

HER2/neu overexpression has been reported in CRC at rates ranging from 2% to 11% similar to that of breast carcinoma. Different immunohistochemistry (IHC) markers, subgroups of individuals with varying clinicopathologic CRC characteristics, and scoring systems may all contribute to these disparities. Recent research shows HER2/neu overexpression occurs in approximately 2% of all CRCs and up to 5% in clinical stages three and four with KRAS wild-type tumors [5,11,12].

HER2/neu interpretation in colorectal carcinoma

Despite the frequent use of IHC in carcinomas of breast and stomach, IHC as well as fluorescence in situ hybridization (FISH) have not yet been completely adapted for HER2/neu amplification in CRC [13,14]. The guidelines for immunohistochemistry/fluorescence in situ hybridization interpretation in CRC are summed up in Table 1.

**Table 1:** Guidelines of HER2/neu interpretation in colorectal carcinomas. IHC: immunohistochemistry; HER2/neu: human epidermal growth factor receptor 2; HERACLES: HER2 amplification for colorectal cancer enhanced stratification; FISH: fluorescence in situ hybridization; CEP-17: centromere enumerator probe 17

Tumor site	Negative	Equivocal	Positive
Colorectal cancer VENTANA (based on Ruschoff et al.) [13]	IHC score 0: no staining or in less than 10% of tumor cells, granular or segmental.	IHC score 2+: weak to moderate complete, circumferential, basolateral, or lateral membrane reactivity in >10% of tumor cells.	IHC score 3+: intense staining in >10% cells, circumferential, basolateral, or lateral.
	IHC score 1+: incomplete faint membranous staining more than 10%.	FISH mandatory	FISH not mandatory
	FISH score: <4 copies or HER2: CEP 17 (<1.8).	FISH score: 4-6 copies or HER2/neu: CEP 17 (1.8-2) in >10% of cells.	FISH score: >6 copies or HER2: CEP 17 (>2) in >10% of cells.
Colorectal cancer HERACLES (Valtorta et al.) [11]	IHC score 0: no staining.	IHC score 2+: moderate staining in 50% of cells.	IHC score 3+: intense staining in more than 50% of cells.
	IHC score 1+: faint membranous staining (granular or segmental); moderate staining in <50% of cells; intense staining <10% of cells.	FISH mandatory: if more than 50% cellularity is confirmed following retesting of IHC ISH; HER2/neu: CEP 17 (>2) in >50% of cells.	FISH is not mandatory: if intense staining in more than 10% but less than 50% of cells.
			FISH mandatory: if more than 10% cellularity is confirmed following retesting of IHC ISH; HER2/neu: CEP 17 (>2) in more than 50% of cells.

Valtorta et al. in 2015 devised IHC/FISH criteria for HER2/neu overexpression in CRC and identified patients for enrolment in the phase II HER2 amplification for colorectal cancer enhanced stratification (HERACLES) trial. HER2 expression analysis by immunohistochemistry was performed manually using HercepTest antibody (Glostrup, Denmark: Dako A/S) and automatically on the automated BenchMark ULTRA system (Oro Valley, AZ: Roche Tissue Diagnostics) using the VENTANA 4B5 antibody, following the manufacturers’ instructions in both cases. ERBB2 amplification analysis by FISH was performed with a PathVysion HER-2 DNA Probe Kit (Des Plaines, IL: Abbott Laboratories) [11].

According to the HERACLES Diagnostic Criteria, 5% of KRAS wild-type advanced CRC patients exhibited HER2/neu-positive tumors in both the archival and clinical validation cohorts. Relative to prior reports, current CRC investigations found HER2/neu positivity (IHC 2+/3+) ranged from 1.6% to 6.3%. HER2/neu amplification rates in CRC range from 1.8% to 22% using molecular testing by next-generation sequencing (NGS) and clinical genome sequencing (CGS) platforms [12,15].

Additional approaches to detect HER2/neu using liquid biopsies were first investigated in patients with breast cancer and recently in metastatic CRC (mCRC) [16]. Takegawa et al. studied ctDNA in 18 patients with cetuximab-resistant mCRC, four of whom (22%) were HER2/neu positive [17]. Rebiopsy of any of these four patients with metastatic lesions showed HER2/neu amplification in both plasma and tissue samples. HER2/neu activating mutations or amplifications were discovered in 4% of the 143 CRC patients studied by Schrock et al. [18]. IHC is widely available and has been used successfully in therapeutic HER2/neu trials.

HER2/neu-positive CRC pathology

Both the sides of the colon and rectal tumors have well-defined epidemiology, pathology, mutation profile, and presentation. Proximal tumors are more prone to hypermethylation as well as to possess microsatellite instability (MSI) when compared to distal tumors. According to recent meta-analyses, the survival of mCRC tumors on the right in comparison to the left side is significantly worse [19,20]. Several CRC studies reported differential expression based on tumor characteristics, location, and histological characters. On the other hand, HER2/neu and EGFR amplified cancers in the distal tissues (splenic, colonic, and rectal) were more common in the Pan-European Trials in Alimentary Tract Cancer (PETACC)-3 trial [21,22].

In the HERACLES-A trial, 64% of distal tumors and 21% of rectal tumors were HER2/neu-positive mCRC [11]. It was discovered in 4.3% of sufferers with increased risk, locally advanced rectal tumor who were subjected to chemoradiation with or without cetuximab in phase II EXPERT-C study [23]. Marshall et al. reported a 5.4% of HER2/neu positivity for rectal carcinomas (Table 2) [24].

**Table 2:** Prevalence of HER2/neu positivity in patients with colorectal cancer. HER2/neu: human epidermal growth factor receptor 2

Study (year)	Location	Number of cases	HER2/neu negative % (0/1+)%	HER2/neu positive % (2+/3+)%
Xu et al. (2015) [25]	China	717	55	45
Torabizadeh et al. (2016) [26]	Iran	50	60	40
Shabbir et al. (2016) [27]	Pakistan	95	45	55
Sawada et al. (2018) [28]	Japan	359	95.9	4.1
Kamal and Jalal (2019) [29]	Erbil/Kurdistan	103	46.6	53.4
Benli and Barut (2020) [30]	Turkey	123	66	34

HER2/neu in CRC prognosis

HER2/neu’s prognostic role in CRC is not completely known. HER2/neu amplification was previously thought to be correlated with a bad prognosis but recent research found no such link. However, in a large study cohort of 1645 patients having stage I-IV CRC, patients with HER2/neu-positive tumors had worse overall survival (OS) than those with negative HER2/Neu tumors [7].

The PETACC-8 study found HER2/neu amplification to be a poor prognostic indicator in stage III colon carcinoma patients. HER2/neu mutations were found in 66/1689 patients (3.9%). Shorter time to response (TTR) (hazard ratio {HR} 1.9, 95% confidence interval {CI} 1.1-3.2) and OS (HR 1.7, 95% CI 0.9-3.2) were linked to HER2/neu amplification and mutation by NGS and FISH, respectively. Age, therapy, grading, tumor locality, pathological stage and node status, perforation of the colon, and vascular or lymphatic’s invasion were all taken into account in the analysis. The low incidence of HER2/neu amplifications in CRC makes it difficult in assessing their potential prognostic effect, possibly explaining the mixed results of the studies. Despite this, the data suggest that HER2/neu has a less severe prognostic impact than other uncommon mutations like BRAF [8].

HER2/neu as a new CRC therapeutic target

HER2/neu was studied as a curative select in mCRC for over a decade with mixed results. Trastuzumab plus folinic acid, fluorouracil and oxaliplatin (FOLFOX) therapy was evaluated as a second- or third-line treatment for mCRC in a phase II study. The patients who were IHC proven HER2/neu (2+) tumors which are considered equivocal were eligible and no FISH testing was planned. Twenty-six (4%) of 653 screened tumor blocks had HER2/neu 2+. Five (24%) of the 21 evaluable patients had a limited reaction. The little HER2/neu positivity rate prevented the completion of the trial [31].

Trastuzumab plus irinotecan in first- or second-line advanced CRC with HER2/neu overexpression was also studied in nine patients. In 11 (8%) of 138 tested tumors, IHC detected HER2/neu 2+ in five patients and HER2/neu 3+ in six patients. Five of seven patients (71%) had partial responses, four of which lasted six weeks. The median was 14 months [32]. Capecitabine, oxaliplatin, and lapatinib were also reported to have helped two patients who have mCRC with liver metastases, but their HER2/neu status was not known [33].

Efficacy data were inconclusive and enrollment was low in such studies, most likely because of the absence of a mechanistic-based HER2/neu targeted preclinical strategy, fixed sample size based upon the predicted occurrence of amplification of HER2/neu in the study population. Also, in some studies, concomitant chemotherapy makes it difficult to govern the role of HER2/neu blockade in the therapeutic outcome [15].

Utilizing xenografts, Bertotti et al. discovered an effective treatment target for cetuximab-resistant CRC amplified HER2/neu from patients with mCRC. HER2/neu amplified mCRC xenografts were sensitive to trastuzumab plus lapatinib, but not either agent alone. It was based on these preclinical findings that the HERACLES studies were initiated [34].

In phase II HERACLES study from Sartore-Bianchi et al., HER2/neu-positive patients and who were already been given an average of four previous lines of therapy were prescribed oral lapatinib 1000 mg/day and trastuzumab 2 mg/kg weekly. A total of 74% of patients attained disease control rate (DCR), while 30% of patients attained overall response rate (ORR) [12].

The MOUNTAINEER study is an ongoing single-arm phase II trial testing tucatinib, an oral selective small molecule inhibitor of HER2/neu receptor in metastatic colorectal carcinoma patients and is given 300 mg/day combined with trastuzumab 6 mg/kg every three weeks [35]. Patients with HER2/neu positive mCRC have shown clinical improvement from anti-HER2/neu therapy, according to the published case reports (Table 3) [36-38].

**Table 3:** Clinical studies using HER2/neu as a target for mCRC. HER2/neu: human epidermal growth factor receptor 2; mCRC: metastatic colorectal carcinoma; RAS: rat sarcoma virus; HERACLES: HER2 amplification for colorectal cancer enhanced stratification; FISH: fluorescence in situ hybridization; NGS: next-generation sequencing

Reference	HER2/neu expression %	Treatment	Response rate	Status
Ramanathan et al. [32]	3.6 (2+)	Trastuzumab and irinotecan	71%	Published
HERACLES-A; Siena et al. [15]	21 (2+) 79 (3+)	Trastuzumab and lapatinib	30.3%	Published
Clark et al. [31]	4 (2+, 3+)	5FU, leucovorin, oxaliplatin, and trastuzumab	24%	Published
HERACLES-rescue [15]	100	Trastuzumab and pertuzumab	38.2%	Published
MOUNTAINEER [35]	HER2/neu positive, FISH or NGS, RAS wild-type	Tucatinib and trastuzumab	Awaited	Ongoing

## Conclusions

In spite of the improvements made in the therapies of CRC, till date no specific oncotargets beyond targeting RAS and BRAF genes are available. Due to its histological diverseness, CRC is one of the most important fields for developing onco-targeted therapies. On the basis of the obtainable data, HER2/neu overexpression seems to be a clinically applicable molecular feature for a subgroup of patients with CRC. Its anticipating role combined with anti-EGFR therapy shows prognostic importance. After introducing the HERACLES diagnostic criteria for HER2/neu overexpressing cancers and integrating conventional procedures like IHC and FISH with newer techniques like NGS as well as CGS together make an accurate demonstration of HER2/neu status in CRC.

Despite the fact that phase III trials with HER2/neu targeted therapies are yet to come, it is safer to say several HER2/neu targeted therapies are already being considered for the role of anti-HER2/neu therapy. Due to their appropriate action at the therapeutic level along with compelling comparison with other medicine approaches, this may lead to its inclusion in the treatment programs providing a novel alternative for refractory cases to conventional treatment where outcomes remain still unfavorable.
